# Stem cell therapy offers new hope for the treatment of Alzheimer’s disease

**DOI:** 10.3389/fcell.2025.1650885

**Published:** 2025-08-14

**Authors:** Guodong He, Jingnan Huang, Zhaodi Zeng, Huiyu Sun, Chao Wu, Qi Xu, Chuanchen Hu, Bei Jin, Minfeng Tong, Chengde Wang

**Affiliations:** ^1^ Department of Neurosurgery, Affiliated Jinhua Hospital, Zhejiang University School of Medicine, Jinhua, Zhejiang, China; ^2^ Department of Neurosurgery, Wencheng Hospital Affiliated of Wenzhou Medical University, Wenzhou, Zhejiang, China; ^3^ Department of Paediatrics, Wencheng Hospital Affiliated of Wenzhou Medical University, Wenzhou, Zhejiang, China; ^4^ Department of Traditional Chinese Medicine and Gynaecology, Hangzhou Hospital of Traditional Chinese Medicine, Hangzhou, Zhejiang, China; ^5^ Department of Rehabilitation, Affiliated Jinhua Hospital, Zhejiang University School of Medicine, Jinhua, Zhejiang, China; ^6^ Department of Neurology, Huzhou Hospital Affiliated of Zhejiang University, Huzhou, Zhejiang, China; ^7^ Department of Neurology, Affiliated Jinhua Hospital, Zhejiang University School of Medicine, Jinhua, Zhejiang, China; ^8^ Department of Neurosurgery, The First Affiliated Hospital of Wenzhou Medical University, Wenzhou, Zhejiang, China

**Keywords:** alzheimer’s disease, stem cells, regenerative therapy, paracrine and immunomodulatory mechanisms, amyloid-β clearance

## Abstract

Alzheimer’s disease (AD) is a progressive neurodegenerative disorder primarily characterized by memory impairment and cognitive decline, for which no curative treatment is currently available. Existing therapeutic strategies, such as cholinesterase inhibitors and N-methyl-D-aspartate (NMDA) receptor antagonists, can only provide limited symptomatic relief and fail to halt disease progression. In recent years, stem cell therapy has emerged as a promising approach for AD due to its multifaceted mechanisms of action. The therapeutic effects of stem cells in AD are mainly attributed to their ability to differentiate into functional neurons or glial cells, thereby replacing damaged cells and repairing neural networks. In addition, stem cells secrete neurotrophic and anti-inflammatory factors that contribute to the improvement of the brain microenvironment. Furthermore, they can regulate neuroinflammation, promote the clearance of β-amyloid (Aβ) deposits, and suppress neuroinflammation, thus potentially slowing disease progression. However, several challenges remain, including low cell survival rates, immune rejection, tumorigenic risks, and difficulties in crossing the blood-brain barrier. Looking ahead, the integration of advanced technologies such as organoid models, gene editing, artificial intelligence, and multi-omics approaches may drive substantial progress in the clinical translation of stem cell therapies for AD. Although still in its early stages, the future of this therapeutic strategy holds great promise.

## Introduction

Alzheimer’s disease (AD) is a progressive neurodegenerative disorder primarily characterized by memory decline, cognitive impairment, and behavioral dysfunction (Kn et al., 2021). It significantly compromises patients’ quality of life and imposes a substantial burden on social care systems. Its core pathological features include the deposition of β-amyloid (Aβ) plaques, tau protein hyperphosphorylation leading to neurofibrillary tangles, synaptic loss, and neuroinflammation (Chen and Yu, 2023). Although significant progress has been made in understanding the pathogenesis of AD, current clinical treatments—such as cholinesterase inhibitors (e.g., donepezil) and NMDA receptor antagonists (e.g., memantine)—only provide symptomatic relief without halting or reversing disease progression (Li et al., 2024). Recent clinical trials have demonstrated that anti-Aβ monoclonal antibodies (such as aducanumab), though capable of reducing cerebral Aβ burden, exhibit controversial clinical benefits and are often associated with serious adverse events (Villain et al., 2025; Petersen et al., 2025). Consequently, there is an urgent need to develop novel therapeutic strategies that target the underlying pathology of AD through fundamentally different mechanisms.

As a breakthrough in the field of regenerative medicine, stem cell therapy holds great promise for the treatment of neurological disorders due to its unique capabilities in cell replacement, paracrine regulation, and immunomodulation. Stem cells can differentiate into functional neurons or glial cells, thereby replacing damaged cells and reconstructing neural networks (Selvaraj et al., 2012; Bai et al., 2023). Moreover, they secrete neurotrophic factors and anti-inflammatory cytokines that modulate the brain microenvironment, promote neuroregeneration, and facilitate synaptic remodeling (Li et al., 2025; Mukhamedshina et al., 2019; Li et al., 2022; Müller et al., 2021). In addition, certain types of stem cells, such as mesenchymal stem cells (MSCs), can modulate microglial phenotypes and enhance their phagocytic capacity toward Aβ, thus exerting a multi-targeted intervention on the pathological progression of AD (Shin et al., 2014).

Although stem cell therapy has demonstrated significant efficacy in animal models of AD, its clinical translation still faces numerous challenges, including low cell survival rates, immune rejection, potential tumorigenicity, and delivery barriers (Marei, 2025). To overcome these obstacles, researchers are increasingly incorporating advanced technologies such as gene editing, exosome-based delivery, organoid modeling, and multi-omics analyses to enhance therapeutic efficacy and safety.

This review systematically elucidates the underlying mechanisms, recent research progress, ongoing clinical trials, and key challenges in the clinical application of stem cell therapy for Alzheimer’s disease, with a particular emphasis on its integration with precision medicine and individualized therapeutic strategies.

### Pathogenesis and current therapeutic strategies of Alzheimer’s disease

**FIGURE 1:**
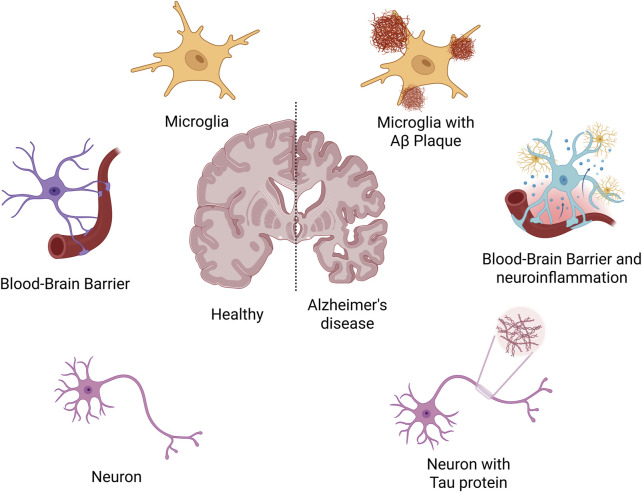
The pathogenesis of Alzheimer’s disease.

AD is a progressive neurodegenerative disorder characterized by cognitive decline and memory impairment (Kn et al., 2021; Breijyeh and Karaman, 2020). Its pathogenesis is multifactorial, involving the interplay of several pathological mechanisms (Figure 1). A central feature is the abnormal accumulation of Aβ peptides, particularly Aβ42, which aggregates into soluble oligomers and insoluble plaques (Ding et al., 2025; Takahashi et al., 2017; Kocahan and Doğan, 2017). These deposits exert direct neurotoxic effects and activate glial cells, inducing chronic neuroinflammation and oxidative stress, thereby disrupting synaptic function and neural network stability. Concurrently, hyperphosphorylation of Tau protein leads to the formation of neurofibrillary tangles, impairing axonal transport and promoting neuronal apoptosis (Liu et al., 2020; Zhang et al., 2009). Synaptic dysfunction and neuronal loss emerge early in the disease course and are directly responsible for cognitive deterioration. Moreover, deficits in the central cholinergic system, including reduced acetylcholine synthesis, further impair attention and memory processing (Herholz et al., 2008; Maurer and Williams, 2017; He et al., 2023). In addition, sustained release of pro-inflammatory cytokines including TNF-α and IL-1β exacerbates neuronal damage and compromises the blood-brain barrier (Galea, 2021). Oxidative stress, often driven by mitochondrial anomalies resulting in increased reactive oxygen species (ROS) production, contributes to widespread cellular injury (Cheignon et al., 2018). Genetic factors, such as the presence of the APOE ε4 allele and mutations in APP, PSEN1, and PSEN2, also play a critical role in disease susceptibility by affecting Aβ metabolism and immune responses (Almkvist and Graff, 2021; Behl and Behl, 2023).

Despite extensive research, effective disease-modifying therapies (DMTs) for AD remain very limited. Currently, AD treatment mainly focuses on symptomatic management, supplemented by DMTs and various supportive interventions, aiming to delay disease progression, improve cognitive function, and enhance quality of life (Nawaz et al., 2025; Zh et al., 2025; Wang et al., 2024). The most commonly used drugs are cholinesterase inhibitors (donepezil, rivastigmine, galantamine), which improve early cognitive symptoms by inhibiting acetylcholine breakdown, and the NMDA receptor antagonist memantine, which reduces glutamate-mediated excitotoxicity in moderate to severe cases (Grossberg, 2003; Nafees et al., 2021; K et al., 2024). Although these drugs offer short-term symptom relief, they do not prevent neurodegeneration and have limited efficacy with notable side effects. Recently, monoclonal antibodies targeting Aβ, such as aducanumab and lecanemab, have demonstrated potential in reducing amyloid pathology and received FDA accelerated approval; however, their cognitive benefits remain controversial and they carry safety risks like cerebral edema (Cummings, 2025). DMTs mainly target Aβ and Tau proteins, but anti-Tau therapies are still in early development, and many approaches like BACE1 inhibitors or neuroinflammation modulators have been discontinued due to limited efficacy or safety concerns (Iwatsubo, 2024). Beyond pharmacological treatment, non-drug interventions—including cognitive training, exercise, Mediterranean diet, psychological support, and multisensory therapy—play a vital role in comprehensive AD management by delaying functional decline, improving behavioral symptoms, and enhancing overall patient wellbeing (Li X. et al., 2023; Barros-Aragão et al., 2025; Liu et al., 2025; Soni et al., 2025).

Although traditional treatments can alleviate cognitive symptoms and slow the progression of AD to some extent, their efficacy is limited, they cannot reverse pathological changes, and they are often accompanied by adverse effects. In particular, given the complex and multifaceted pathological mechanisms of AD, conventional therapies usually target a single pathway, making it difficult to comprehensively regulate the disease process. Therefore, researchers have increasingly turned their attention to emerging therapies with multifunctional and regenerative potential—such as stem cell therapy—aimed at opening novel avenues for AD treatment through mechanisms including cell replacement, immunomodulation, and neural repair.

### Types and characteristics of stem cells

Stem cells currently explored in AD research and potential therapeutic applications mainly include neural stem cells (NSCs), mesenchymal stem cells (MSCs), and induced pluripotent stem cells (iPSCs). These stem cell types differ in their sources, differentiation potential, biological functions, and clinical application prospects. Each has demonstrated significant value in various aspects of AD research, including mechanistic studies, animal model development, drug screening, and potential therapeutic interventions.

### Neural stem cells (NSCs)

NSCs are characterized by their multipotency, allowing them to both self-renew and differentiate into neuronal and glial lineages, including neurons, astrocytes, and oligodendrocytes (Eckert et al., 2017). They possess an inherent capacity for lineage-specific differentiation within the nervous system. Numerous studies have demonstrated that transplanted NSCs can survive long-term in brain regions closely associated with cognitive function, such as the hippocampus and cortex, where they are capable of forming synaptic connections with host neurons and integrating into existing neural networks (Vishwakarma et al., 2014; Anderson, 2001; Zhao and Moore, 2018). This integration contributes to partial restoration of cognitive function and improvement in learning and memory deficits. In addition, NSCs produce a range of neurotrophic molecules, notably brain-derived neurotrophic factor (BDNF) and nerve growth factor (NGF), which support endogenous neurogenesis and synaptic remodeling, thereby helping to improve the impaired neural microenvironment observed in AD (L et al., 2021; Hsu et al., 2007; Salgado et al., 2015). Although current research on NSCs remains largely at the preclinical animal study stage, their multifaceted roles in structural repair and restoration of function establish a promising basis for the future application of cell-based replacement therapies in AD.

### Mesenchymal stem cells (MSCs)

MSCs are a type of adult stem cell with broad tissue sources, ease of acquisition, and minimal ethical concerns (Zhou and Shi, 2023). These cells can be obtained from bone marrow sources, umbilical cord, adipose tissue, and other sources. Owing to their strong immunomodulatory capacity, anti-inflammatory properties, and robust paracrine activity, MSCs have emerged as promising candidates for AD therapy. Studies have shown that MSCs can modulate the central immune microenvironment by promoting the polarization of microglia from the pro-inflammatory M1 phenotype to the anti-inflammatory M2 phenotype, thereby achieving the anti-inflammatory objective (Kråkenes et al., 2024). In addition, MSC-secreted cytokines and growth factors promote synaptogenesis and axonal repair, helping to alleviate neurodegeneration. Notably, MSCs also release exosomes—nano-sized vesicles enriched with functional RNAs (e.g., miRNAs, lncRNAs) and proteins—which can cross the blood-brain barrier and persist in the nervous system (Chang et al., 2024; Ye et al., 2023; Feizi et al., 2024). These exosomes exert therapeutic effects by regulating gene expression, suppressing neuroinflammation, and promoting neuroprotection, indicating a hopeful path toward cell-free therapeutic intervention. The efficacy of MSCs has been extensively validated in animal models, providing strong support for their future clinical translation.

### Induced pluripotent stem cells (iPSCs)

**FIGURE 2:**
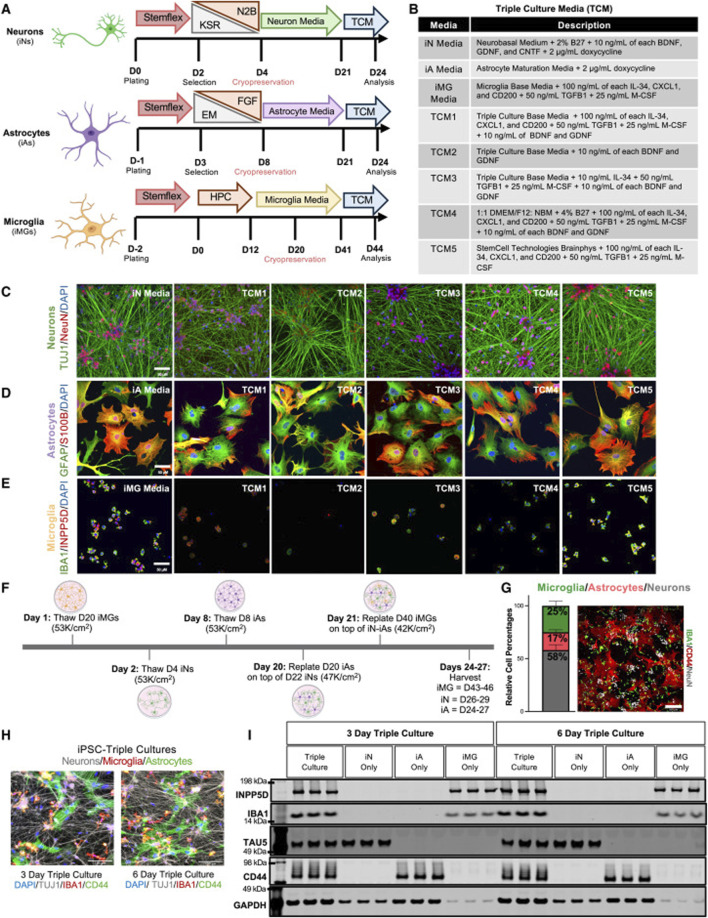
Selection of the optimal media for triculture of human iPSC-derived neurons, astrocytes, and microglia **(A)** Schematic of the iPSC differentiation protocols for neurons (iNs), astrocytes (iAs), and microglia (iMGs). iNs and iAs were generated by lentiviral expression of lineage-specific transcription factors, while iMGs were derived via a hematopoietic precursor (HPC) stage using non-viral methods. Cryopreservation days are indicated; each cell type was fully differentiated before switching to triculture media (TCM). Key abbreviations: KSR, knockout serum replacement; N2B, neu robasal with N2/B27; EM, expansion medium; FGF, fibroblast growth factor medium. **(B)** Table summarizing the composition of each cell-type-specific media and TCM. **(C–E)** Representative immunostaining of neurons **(C)**, astrocytes **(D)**, and microglia **(E)** maintained in either their respective media or TCM. Markers shown include TUJ1/NeuN for neurons, GFAP/S100B for astrocytes, and IBA1/INPP5D for microglia. Scale bars, 50 μm. **(F)** Timeline of the triculture workflow. Cryopreserved iNs, iAs, and iMGs were thawed and matured separately; astrocytes and microglia were sequentially plated onto neuron cultures on days 20 and 21 and then co-cultured for 3–6 days. The days in bold refer to the start of thawing the first stock of cryopreserved cells, and the non-bolded days refer to the day of differentiation for each cell type. **(G)** Bar plot showing the relative percentages of NeuN + (neurons), IBA1 + (microglia), and CD44 + (astrocytes) cells at day 27, determined from immunostaining (n = 2 genetic backgrounds, three differentiations, and two wells per differentiation). Error bars represent standard error. A representative field of view (FOV) is shown, with six FOVs per well analyzed by blinded quantification. Scale bar, 200 μm. **(H)** Representative triculture images (3–6 days of co-culture) labeled for CD44 (astrocytes), IBA1 (microglia), and TUJ1 (neurons). Scale bars, 100 μm. **(I)** Western blot of tricultures at days 3 and 6, probed for INPP5D, IBA1, TAU5, CD44, and GAPDH, confirming the presence of all 3 cell types (Lish et al., 2025)

Since Takahashi and Yamanaka first induced pluripotent stem cells from mouse fibroblasts in 2006, this cell reprogramming technology has remained a hot topic of research (Yamanaka and Takahashi, 2006). iPSCs are reprogrammed cells with pluripotency similar to embryonic stem cells, generated via the induction of key transcriptional regulators such as Oct4, Sox2, Klf4, and c-Myc—into adult somatic cells, such as dermal fibroblasts (Zhang et al., 2021; Arrighi and Arrighi, 2018). iPSCs possess unlimited self-renewal capacity and can differentiate into various functional cell types, including neural cells. In AD research, iPSCs can be directionally induced *in vitro* to generate specific neuronal subtypes, such as cholinergic neurons, which may replace the large number of neurons lost due to disease progression, thereby offering substantial potential for regenerative therapy (Wang J. et al., 2025). Moreover, iPSCs provide a valuable platform for patient-specific disease modeling. By reprogramming somatic cells from AD patients, iPSCs can replicate early pathological events of the disease, facilitating the study of disease mechanisms and the screening of new therapeutic agents. It is worth mentioning that Lish et al. developed a co-culture system of neurons, astrocytes, and microglia based on traditional iPSCs and discovered that disease-related microglia may be beneficial in the early stages of familial AD (Figure 2) (Lish et al., 2025). However, the clinical application of iPSCs still faces several challenges, including high cellular heterogeneity, unstable differentiation efficiency, and potential tumorigenicity (Moy et al., 2023). These issues call for further advancements in genetic safety control, optimized differentiation protocols, and transplantation strategies.

## Mechanisms of stem cell therapy in the treatment of Alzheimer’s disease

### Cell replacement

One of the hallmark features of AD is the widespread loss of neurons, particularly cholinergic neurons, which is closely associated with cognitive impairment (Ahmed et al., 2017; Ferreira-Vieira et al., 2016). The cholinergic system, especially the neuronal populations originating from the basal forebrain, plays a critical role in learning and memory. Dysfunction in this system is considered a key contributor to AD pathogenesis (Wei et al., 2024; Wang Z. et al., 2025; Wang et al., 2020). Although traditional therapies such as cholinesterase inhibitors can temporarily increase acetylcholine levels, they do not halt the progressive loss of neurons (Grossberg, 2003; Khanam et al., 2016). Consequently, recent research has focused on cell replacement therapies aimed at replenishing or reconstructing lost neuronal populations to restore neural network function (Telias and Ben-Yosef, 2015).

iPSCs and NSCs serve as important cellular sources for AD cell replacement strategies (Yang et al., 2016a; Al Abbar et al., 2020). iPSCs possess pluripotency and can differentiate into virtually any neural cell type, whereas NSCs, with their inherent neuroectodermal lineage bias, more readily give rise to neurons and glial cells (Ardhanareeswaran et al., 2017). Various *in vitro* differentiation protocols have been developed to efficiently induce these stem cells into functional cholinergic neurons. In particular, the introduction of key neurodevelopmental transcription factors such as Neurogenin 2 (NGN2) and LIM homeobox 8 (LHX8) significantly enhances directed differentiation into cholinergic neurons of the cortex and hippocampus (Sun et al., 2001; Liu et al., 2013; Tomioka et al., 2014). These transcription factors are crucial for neuronal lineage commitment, migration, and axonal growth, thereby improving differentiation efficiency toward the cholinergic phenotype.

**FIGURE 3:**
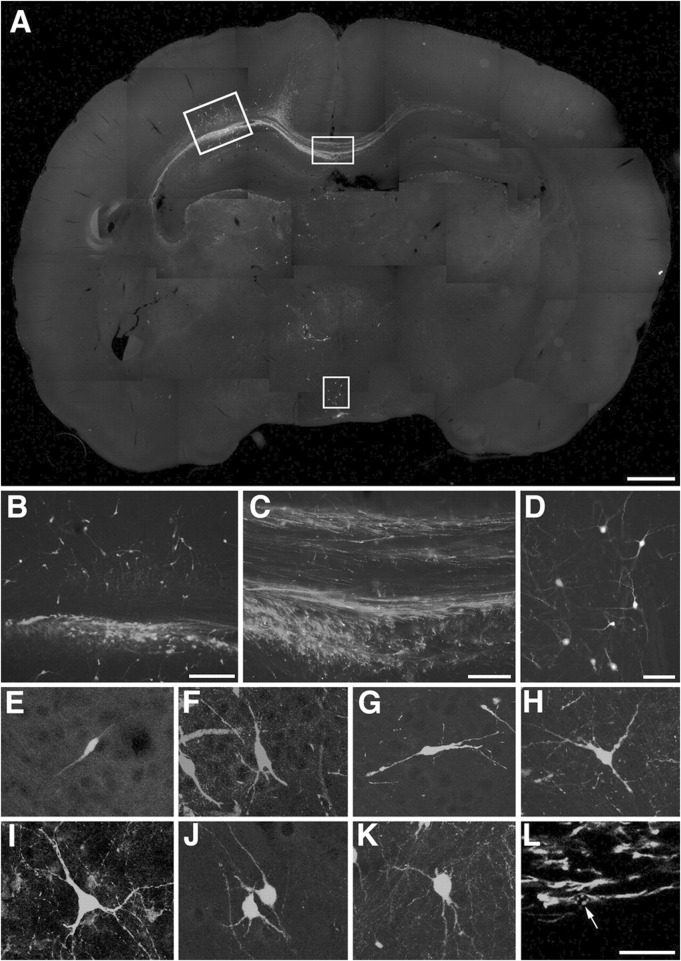
Incorporation of ES cell-derived neurons into the developing rat brain. **(A–K)** Engrafted donor cells identified by their EGFP fluorescence **(E,G–K)** or immunofluorescence with an antibody to EGFP **(A–D,F)** generate a variety of neuronal phenotypes. A, Twenty days after transplantation into the ventricle of E16.5 rats, the cells formed intraventricular clusters and migrated as single cells into various host brain regions. **(B–D)** Higher power microphotographs of areas indicated in **(A)** depicting incorporation into neocortex(B)and hypothalamus(D). Donor-derived cortical neurons were found to extend long axons into the corpus callosum **(C)**. **(E–K)** Confocal microscopy and digital reconstruction revealed that the transplanted cells adopt a variety of morphologies, including simple bipolar cells resembling young migratory neurons (E, neocortex), complex phenotypes mimicking principal pyramidal neurons of the hippocampus (F, CA1pyramidalcelllayer), and multipolar cell types **(G,H)** neocortex; **(I)** septum; **(J)**, thalamus; **(K)**, tectum). **(L)** Immunofluorescence analysis with an antibody to nestin depicts engrafted cells with immature, elongated phenotypes characteristic of migratory precursor cells. The arrow points to the mouse-specific DNA *in situ* hybridization signal used for donor cell identification (tectum, confocal analysis). Scale bars: **(A)** 1 mm; **(B)** 200 m; **(C) (D)** 100 m; **(E–L)** 50 m (Wernig et al., 2004b).

In mouse models of AD, transplantation of these differentiated neurons has shown that they can survive, migrate within the host brain, and form synaptic connections with endogenous neurons (Hayashi et al., 2020; Yang et al., 2016b; Hermann and Storch, 2013). It has confirmed the expression of cholinergic markers such as choline acetyltransferase (ChAT) and synaptophysin, as well as the ability to release acetylcholine (Gu et al., 2015). Moreover, as shown in Figure 3, EGFP fluorescence demonstrates that the transplanted neurons are capable of generating action potentials, suggesting functional integration into neural circuits (Wernig et al., 2004a). Some studies have also reported behavioral improvements in learning and memory tasks in treated animal models, indicating preliminary functional recovery (Kim et al., 2015; Wang et al., 2015).

In addition to replacing cholinergic neurons, stem cells can develop into key neural cell types, including astrocytes and oligodendrocytes. Astrocytes are critical for maintaining the blood-brain barrier, regulating glutamate metabolism, and supporting synaptogenesis; their dysfunction is linked to excitotoxicity in AD (Manu et al., 2023). Oligodendrocytes, responsible for myelin sheath formation, are essential for efficient neural signal transmission, and their loss contributes to cognitive decline (Yeung et al., 2014; Jang et al., 2019). Research has shown that NSCs can differentiate into mature glial cells under appropriate conditions and participate in neural network reconstruction and remyelination following transplantation (Tang et al., 2017; Yamaguchi et al., 2016).

Although cell replacement therapies for AD are still in the experimental stage, their demonstrated neurorestorative effects in animal models provide a strong foundation for future clinical application. Advances in gene editing and tissue engineering may further Improve the accuracy and effectiveness of stem cell therapies targeting Alzheimer’s disease.

### Paracrine effects

In addition to directly replacing lost neurons, another critical mechanism by which stem cell therapy exerts therapeutic effects in AD is through paracrine signaling. Studies have demonstrated that stem cells—particularly mesenchymal stem cells MSCs and NSCs—secrete a variety of bioactive substances, including cytokines, exosomes, and non-coding RNAs, which play significant roles in modulating the neural microenvironment, promoting endogenous neuroregeneration, and alleviating neuroinflammation (Han et al., 2022; Kaminska et al., 2022).

Neurotrophic factors are key components of the paracrine secretome of stem cells. Among them, BDNF, NGF, and glial cell line-derived neurotrophic factor (GDNF) are particularly crucial (Chang et al., 2021; Shen et al., 2019; Allen et al., 2013). These factors not only support neuronal growth and survival but also enhance synaptic plasticity and improve learning and memory capabilities. Research has shown that the transplantation or injection of MSCs and NSCs leads to a significant upregulation of BDNF and other neurotrophic factors in brain tissue, which positively correlates with cognitive improvements.

In addition, stem cells secrete various immunomodulatory factors, such as interleukin-10 (IL-10) and transforming growth factor-beta (TGF-β), which can effectively suppress the expression of pro-inflammatory cytokines like IL-1β and TNF-α (Minev et al., 2024). This anti-inflammatory action mitigates chronic neuroinflammation, a key driver of AD progression that contributes to neuronal apoptosis, synaptic damage, and neural network disruption. The anti-inflammatory effects of stem cells can be further enhanced by promoting the polarization of macrophages or microglia toward the M2 phenotype, which supports both immunosuppression and tissue repair.

**FIGURE 4:**
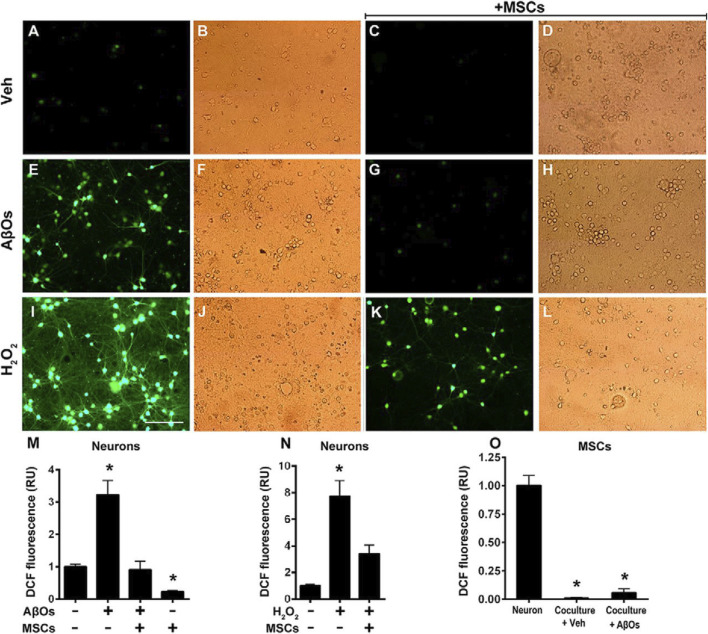
Oxidative stress in hippocampal neurons exposed to AβOs in the absence or presence of MSCs. Photomicrographs showing DCF fluorescence (green) in hippocampal neurons exposed to vehicle **(A–D)** AβOs (500 nM) for **(E–H)** or H2O2 (100 M) for 10min **(I–L)** in the absence or presence of MSCs, as indicated. Scale bar, 100 m. Images were acquired on a Nikon Eclipse TE300 epifluorescence microscope with a 20 objective. Corresponding bright-field images are shown beside each fluorescence image. **(M–O)** quantification of integrated DCF fluorescence intensity normalized by the total number of cells. Panels show integrated fluorescence for AβO-exposed neurons **(M)** H2O2-exposed neurons **(N)** or MSCs cocultured with hippocampal neurons and exposed to vehicle or AβOs, compared with hippocampal neurons alone **(O)**. Data are represented as mean S.E. (error bars) (n 6independentcultures, with triplicate coverslips in each experimental condition); *, p 0.05; two-way ANOVA followed by Tukey’s post hoc test; RU, relative units (de Godoy et al., 2018)

Exosomes released by stem cells are another vital vehicle of their paracrine function. These nanovesicles contain high levels of microRNAs, proteins, and lipids that can be internalized by surrounding neurons or glial cells to modulate gene expression and cellular function. For instance, exosomes derived from MSCs contain miRNAs such as miR-124 and miR-21, which have been shown to regulate inflammation, prevent neuronal apoptosis, and enhance synaptic plasticity—ultimately contributing to protecting hippocampal neurons from oxidative stress damage (Figure 4) (Vakhshiteh et al., 2019; de Godoy et al., 2018; Cheng et al., 2018).

In summary, the paracrine effects of stem cells constitute a major therapeutic mechanism, offering neuroprotective benefits even when the transplanted cells are not fully differentiated or do not persist long-term in the host tissue. Future strategies such as optimizing cell culture conditions, employing genetic modifications, or refining exosome purification techniques may further enhance the paracrine potential of stem cells and improve therapeutic outcomes in AD.

### Pathological clearance

A hallmark of AD pathology is the accumulation of Aβ plaques in brain parenchyma and vasculature, along with hyperphosphorylation of Tau protein leading to the formation of neurofibrillary tangles. These pathological changes disrupt synaptic function, cause neuronal damage and apoptosis, and ultimately result in cognitive decline. Therefore, targeting Aβ deposition and regulating aberrant Tau conformation are crucial therapeutic strategies in AD.

Recent studies have highlighted the potential of stem cells, particularly NSCs and MSCs, in modulating microglial activity and enhancing endogenous clearance mechanisms (Xin et al., 2021). Microglia, the primary immune cells of the central nervous system, display dual phenotypes in AD. The pro-inflammatory M1 phenotype promotes neurotoxicity by releasing cytokines, while the M2 phenotype exhibits enhanced phagocytic activity and tissue repair functions (Orihuela et al., 2016). Stem cells can secrete immunoregulatory cytokines such as TGF-β, IL-4, and IL-10, promoting microglial polarization toward the M2 phenotype (Guo et al., 2022; Kyurkchiev et al., 2014). This transition enhances the clearance of Aβ plaques, reduces local inflammation, and improves the neural microenvironment.

Animal studies have demonstrated a significant reduction in hippocampal Aβ burden following stem cell transplantation, accompanied by behavioral improvements (D'Haese et al., 2020). These effects are not only attributed to immune modulation but may also involve the secretion of Aβ-degrading enzymes such as neprilysin and insulin-degrading enzyme (IDE) by stem cells. Additionally, stem cell-derived exosomes can carry specific microRNAs that regulate the amyloid precursor protein (APP) processing pathway, thereby reducing Aβ production (Huo et al., 2021; Shah et al., 2024).

In summary, stem cell therapy exerts multi-target, integrated regulatory effects on both Aβ and Tau pathology, offering new opportunities for disease-modifying treatments in AD. Future research should focus on elucidating the specific molecular mechanisms involved, particularly those mediated by exosome-based signaling pathways, to facilitate clinical translation.

### Vascular and synaptic remodeling

In addition to neurodegenerative changes, AD is often accompanied by cerebrovascular dysfunction, including cerebral hypoperfusion, capillary degeneration, and blood-brain barrier (BBB) disruption (Love and Miners, 2016). These vascular abnormalities exacerbate Aβ deposition and neuronal damage, creating a vicious cycle. Moreover, synaptic loss and dysfunction are direct causes of cognitive impairment in AD. Studies have demonstrated a strong correlation between synaptic density and cognitive performance, which may even surpass the correlation with Aβ plaque burden (Mecca et al., 2022; DiFilippo et al., 2025). Therefore, vascular repair and synaptic remodeling are critical therapeutic targets of stem cell treatment in AD.

MSCs and NSCs have been shown to secrete various pro-angiogenic factors including vascular endothelial growth factor (VEGF), angiopoietin-1 (Ang-1), and basic fibroblast growth factor (bFGF) (Han et al., 2022; Kniebs et al., 2020). These factors promote capillary regeneration, restore BBB integrity, improve cerebral microcirculation, and enhance local tissue oxygenation and metabolic status. Additionally, TGF-β released by MSCs can inhibit endothelial apoptosis and inflammatory infiltration, thereby slowing cerebrovascular degeneration (de Araújo Farias et al., 2018).

In summary, the combined effects of stem cells on vascular repair and synaptic remodeling provide anatomical and physiological foundations for cognitive restoration in AD. Through multiple coordinated mechanisms, stem cells can modulate the neural microenvironment, offering hope to significantly slow or even reverse neurofunctional decline.

### Progress in animal experiments and clinical studies

Currently, a large body of research on stem cell therapy for AD remains focused on animal experimental stages. The research foundation mainly relies on various classic transgenic AD mouse models, such as APP/PS1 double transgenic mice and 3xTg-AD triple transgenic mice (Sasaguri et al., 2017; Filali et al., 2012). These models exhibit high reproducibility and stability in simulating core pathological processes of AD, such as Aβ plaque formation, phosphorylation of Tau protein, damage to synapses, and cognitive impairment, providing an ideal platform for stem cell therapy research.

Numerous preliminary studies have demonstrated that transplantation of neural stem cells, mesenchymal stem cells, or neurons derived from iPSCs into AD model mice can partially improve cognitive function (Tang et al., 2017; Andrzejewska et al., 2021; Penney et al., 2020). Specifically, treated mice show significant improvements in learning and memory abilities in behavioral tests such as the Morris water maze, Y-maze, and novel object recognition (Qin et al., 2020). Concurrently, there is a marked reduction in Aβ deposition in brain tissue, downregulation of neuroinflammatory cytokines, and partial restoration of synaptic structures in the hippocampus. For example, studies have transplanted neurons induced from human iPSCs into the brains of mice, finding that these cells not only survive and differentiate into mature neurons within the host brain tissue but also functionally integrate with the host neural network, significantly enhancing the ability to learn spatial tasks and recall information assessed by the water maze test (Preman et al., 2021). This further validates the potential of stem cells to reconstruct neural networks and restore function.

Although animal experiments provide a solid theoretical basis and preliminary data support for stem cell therapy in AD, clinical application in humans remains in early exploratory stages, largely confined to Phase I or early Phase II clinical trials (Table 1). Currently, registered or ongoing clinical studies worldwide mainly aim to investigate the safety, feasibility, and early therapeutic outcomes of stem cell treatment. The stem cell types used primarily include autologous bone marrow-derived mesenchymal stem cells (BMSCs) and umbilical cord-derived mesenchymal stem cells (UMSCs).

**TABLE 1:** Clinical trials of stem cell therapy for Alzheimer’s disease.

Stem cell types	NCT number	Source	Trial phase	Research focus
MSCs	NCT01297218	Human umbilical cord blood-derived mesenchymal stem cells	I	Safety and tolerability
MSCs	NCT02833792	Stemedica manufactures allogeneic human mesenchymal stem cells for ischaemic conditions	Ⅱa	Safety and tolerability
MSCs	NCT02600130	Longeveron Mesenchymal Stem Cells)	I	Safety and efficacy
MSCs	NCT02054208	Human umbilical cord blood-derived mesenchymal stem cells	Ⅰ/Ⅱa	Safety and efficacy
MSCs	NCT04040348	Multiple Allogeneic Human Mesenchymal Stem Cells	Ⅰ	Safety and efficacy
MSCs	NCT01547689	Human umbilical cord blood-derived mesenchymal stem cells	Ⅰ/Ⅱ	Safety, tolerability, and efficacy
MSCs	NCT05667649	Autologous adipose-derived stem cells (ADSCs)	Ⅰ	Safety and efficacy

However, it is important to note that current clinical trials face several limitations. First, sample sizes are generally small, with most trials enrolling fewer than one hundred participants, limiting statistical power. Second, follow-up durations are relatively short, often less than 1 year, making it difficult to comprehensively assess long-term efficacy and safety. Third, stem cell sources, dosages, preparation protocols, and infusion routes are not yet standardized, increasing heterogeneity in results. Additionally, patient-related factors such as age, disease stage, and comorbidities may potentially affect responses to stem cell therapy.

Therefore, future work must include large, multicenter RCTs featuring long-term monitoring are essential to validate the therapeutic efficacy of stem cell interventions in AD, clarify their mechanisms of action, safety, and appropriate patient populations, and gradually advance the transition from laboratory research to standardized, individualized clinical application.

### Multidimensional therapeutic decision-making

With ongoing developments in stem cell-based treatments for AD, the introduction of cutting-edge technologies is injecting new vitality into its clinical translation, particularly in terms of safety, efficacy evaluation, and mechanistic exploration.

### Organoid models

Firstly, the construction of brain organoids provides a highly biomimetic platform for preliminary efficacy screening (Chen et al., 2022). These brain-like tissues, self-assembled from human-iPSCs in three-dimensional culture systems, can recapitulate core AD pathological features *in vitro*, such as Aβ plaque deposition, Tau protein aggregation, and synaptic protein loss. This platform serves as a critical bridge between animal models and clinical practice. For example, in the study by Kong et al., brain organoids were used to investigate the causes of neurological deficits induced by SARS-CoV-2 (Kong et al., 2022). Within this system, one can systematically evaluate stem cells from different sources and differentiation stages, as well as combinations of delivery methods and biomaterial scaffolds, thereby optimizing therapeutic protocols and enhancing translational efficiency (Qian et al., 2019; Acharya et al., 2024). Moreover, combined with technologies like single-cell transcriptomics, organoids allow investigation of the interaction mechanisms between transplanted stem cells and host neurons or glial cells, including paracrine signaling pathways, microglial polarization, and cell fusion events, providing high-resolution insights into the underlying mechanisms.

### Artificial intelligence assistance

In personalized therapeutic decision-making, artificial intelligence (AI) is increasingly becoming a vital tool to optimize stem cell treatment strategies (Shende and Devlekar, 2021). Stem cell therapy involves numerous parameters, including cell type, dosage, timing, and target brain regions, which are difficult to comprehensively manage by traditional empirical approaches (Srinivasan et al., 2023). AI models based on deep learning can integrate multimodal data such as MRI/PET radiomics, whole-genome sequencing, and clinical assessment indices to build precise predictive systems. For example, the deep learning model developed by Zhu et al. can quickly and accurately predict the differentiation direction of neural stem cells (Zhu et al., 2021). These systems can forecast treatment responses and evaluate adverse effect risks, assisting clinicians in formulating individualized intervention plans. Additionally, AI techniques can handle large-scale experimental data to decipher regulatory networks controlling stem cell differentiation, and screen potential therapeutic targets and signaling pathways, thereby improving cell engineering efficiency and consistency from the ground up (Li Z. et al., 2023).

In summary, stem cell therapy for Alzheimer’s disease theoretically possesses unique advantages of multitarget and network regulation and has demonstrated remarkable neurorestorative capabilities at the laboratory level. However, bridging the gap from experimental research to clinical application still requires addressing critical issues such as cell stability, safety, clarification of mechanisms of action, and establishment of efficacy evaluation systems. In the future, with the synergistic development of stem cell engineering, neuroimaging technologies, and systems biology, stem cell therapy is expected to become an essential component of precision treatment for AD, bringing breakthrough progress to the field.

## Challenges and prospects

As an emerging regenerative medicine strategy, stem cell therapy has attracted widespread attention in AD research in recent years. Compared with traditional single-target drug interventions, stem cells possess the advantage of multi-mechanism synergistic effects, enabling systemic intervention within the complex pathological context of AD. Although positive progress has been made in mechanistic research of stem cell therapy, its clinical translation still faces multiple challenges.

Despite promising results in preclinical and early-phase clinical studies, the therapeutic outcomes of stem cell interventions in AD are profoundly influenced by patient-specific factors, including age, disease stage, and the presence of comorbid conditions. Advanced age is associated with diminished neuroregenerative capacity and an increasingly hostile brain microenvironment, characterized by chronic inflammation, oxidative stress, and impaired vascular integrity, all of which compromise stem cell survival and differentiation. Moreover, patients at different stages of AD may respond variably to cell-based therapies—while early-stage patients might benefit from neuroprotective and anti-inflammatory effects, advanced-stage patients often suffer from extensive neuronal loss and irreversible structural damage that limits therapeutic efficacy. Comorbidities such as diabetes, cardiovascular disease, and systemic inflammation further exacerbate neurodegeneration and hinder the reparative functions of transplanted stem cells (Evans et al., 2021). These inter-individual variations highlight the necessity of developing personalized or precision medicine approaches. By integrating genomic, epigenetic, imaging, and biomarker data, clinicians can tailor stem cell therapy regimens to patient-specific pathophysiological profiles, thereby optimizing therapeutic efficacy and minimizing risks. Such precision-guided strategies represent a pivotal direction for the future of regenerative therapy in AD.

To accelerate the clinical translation of stem cell therapies for Alzheimer’s disease, several key challenges must be addressed as research priorities. First, large-scale, multicenter, randomized controlled trials (RCTs) with long-term follow-up are essential to generate robust clinical evidence supporting safety, efficacy, and patient selection criteria. Second, advancements in cellular engineering—such as improving stem cell survival, lineage-specific differentiation, and integration into host neural circuits—are critical to enhancing therapeutic precision and consistency. Third, the development of reliable, real-time tracking technologies (e.g., multimodal molecular imaging, nanoparticle labeling) will be pivotal for monitoring stem cell fate, biodistribution, and functional outcomes *in vivo*. Collectively, these strategies must be supported by standardized manufacturing protocols and rigorous quality control frameworks to ensure reproducibility and regulatory compliance. Importantly, stem cell therapy offers a unique therapeutic paradigm in AD by targeting multiple pathogenic processes simultaneously—ranging from cell replacement and immunomodulation to neurotrophic support and synaptic remodeling. This multifaceted and synergistic mechanism distinguishes it from conventional single-target pharmacotherapies and holds immense promise for reshaping the landscape of precision neuroregenerative medicine.

In addition, ethical concerns surrounding the use of induced pluripotent stem cells (iPSCs), including donor consent, genomic integrity, and potential tumorigenicity, alongside regulatory challenges such as the stringent FDA and EMA approval processes and the absence of unified standards for stem cell preparation, underscore the urgent need for globally harmonized guidelines.

In the end, establishing standardized stem cell preparation processes, quality control systems, and multicenter randomized controlled clinical trials will be crucial to promote clinical implementation. More and better-designed preclinical trials are necessary to evaluate the therapeutic effects of stem cells from different sources on AD, with careful exploration of stem cell dosage, long-term safety, efficacy, and precise mechanisms of action. Currently, stem cell therapy for AD is still in its infancy, but it holds great promise to bring more breakthroughs in the future.
